# Perceptual-cognitive three-dimensional multiple-object tracking task can help the monitoring of sport-related concussion

**DOI:** 10.1136/bmjsem-2018-000384

**Published:** 2018-10-01

**Authors:** Jean-François Chermann, Thomas Romeas, Flore Marty, Jocelyn Faubert

**Affiliations:** 1 Consultation commotion et sport, 2, rue de la convention, Paris, France; 2 Sport sciences, Institut National du Sport du Québec, Montreal, Quebec, Canada; 3 School of optometry, Université de Montréal, Montreal, Quebec, Canada

**Keywords:** concussion, sporting injuries, rugby, trauma, neurology, 3D-MOT, return to play, perceptual-cognitive skills

## Abstract

**Objectives:**

While the rate of sport-related concussion is increasing, more effective tools are needed to help monitor the diagnosis and return to play of athletes. The three-dimensional multiple-object tracking (3D-MOT) exercise is a perceptual-cognitive task that has shown predictive power towards the dynamic requirements of real-world activities such as sport. This study introduced the use of the 3D-MOT task, along with the Standardized Assessment of Concussion (SAC) and Modified Balance Error Scoring System (M-BESS) tests, for diagnosis and return to play in professional sports.

**Methods:**

Fifty-nine professional athletes were tested with the 3D-MOT, SAC and M-BESS tests at 48 hours following the injury. The same measures were employed to evaluate the return to play following the standard concussion management protocol. The SAC and M-BESS tests were also performed in pre-season (baseline) in 32 out of the 59 athletes.

**Results:**

The injured athletes exhibited poor performance on 3D-MOT at 48 hours post injury compared with return to play (p<0.001) as well as compared with healthy professionals’ performance scores (p<0.001). Importantly, learning rate, which participants are thought to have an expert advantage on this perceptual-cognitive task, was totally disrupted at 48 hours post injury compared with healthy professionals (p<0.001). The 3D-MOT performance was also correlated to the total number of symptoms (p=0.020), SAC (p=0.031) and M-BESS (p=0.004) scores at 48 hours. Not surprisingly, SAC and M-BESS tests’ usefulness for monitoring concussion was found to be weak, particularly when test performance following the injury was compared to baseline (p=0.056 and 0.349 for SAC and M-BESS, respectively).

**Conclusion:**

3D-MOT could help monitor sport-related concussion in professional athletes. The discussion also covers the critical importance of perceptual-cognitive assessment following concussion in the athletic population.

**What are the new findings?**

- Three-dimensional multiple-object tracking (3D-MOT) processing and learning abilities are disrupted following concussion (48 hours).
- 3D-MOT performance is correlated to the total number of symptoms, Standardized Assessment of Concussion and Modified Balance Error Scoring System tests performance at 48 hours.
- 3D-MOT is useful to assess and monitor athletes’ perceptual-cognitive capacity following concussion.
- Perceptual-cognitive skills should be evaluated following sport-related concussion.

## Introduction

Sport-related concussion, also referred to as mild traumatic brain injury (mTBI), is *‘*induced by biomechanical forces following an impulse force transmitted to the head’ as defined by the fifth consensus statement on concussion in sport.^1^ Over the past two decades, a high number of sport activities were associated with an increased risk and rate of concussion.^2 3^ For instance, rugby is a high contact sport where the incidence of concussion varies from 0.62 to 9.05 per 1000 hours of play, with a higher risk in professional players.^4–7^ A recent study reported rugby with the highest incidence rate of mTBI by far in youth sports, among other contact sports such as football (7.8 times higher) or hockey (3.5 times higher).^8^

The main problem with mTBI is that patients are often considered to be ‘normal’ on neurological examinations or standard clinical testing and are discharged from follow-up programme. Even worse, <10% of sports-induced concussions result in a loss of consciousness^9^ which consequently results in the trauma either not being diagnosed or concealed by the player who does not wish to be sidelined. To help healthcare providers during the initial diagnosis of the concussion and management of the decision to return to play (RTP), the Concussion in Sport Group consensus statement provides updated guidelines including the Sport Concussion Assessment Tool (SCAT).^1^ The SCAT is a concussion evaluation tool relying on several subsets including cognition, balance and symptom evaluation assessed respectively with the Standardized Assessment of Concussion (SAC), the Modified Balance Error Scoring System (M-BESS) and a symptom checklist.^10^ Normative data and concussion cut-off scores have recently been published.^11 12^ However, the reliability and repeatability of these standard clinical tests are still debated.^13–18^ Therefore, more sensitive clinical tools are still required for the diagnosis and the management of sport-related concussion.

A key performance indicator in sport is an athlete’s perceptual-cognitive capacity, which is highly solicited due to the dynamic and time constraints of their changing environment.^19^ This ability represents the human brain’s capacity to extract meaningful contextual information from the dynamic visual scene and is reflected on the field by anticipation and decision-making skills.^20^ Therefore, it appears critical to include an evaluation of ones perceptual-cognitive capacity while monitoring their return to sport activities. In this regard, the three-dimensional multiple-object tracking (3D-MOT) exercise is proposed. This context-free task involves the processing of a visual dynamic scene reflecting some of the fundamental demands required during sport (eg, keeping track of teammates, opponents and the ball) or daily life activities (eg, driving, walking in a crowd).^21 22^ The test requires the participant to process complex motion using selective, sustained and distributed attention as well as working memory.^21 23^ It has been widely used to assess the human perceptual-cognitive capacity in sport but also in other domains such as driving or flying.^22 24–27^ Importantly, in a cohort of 304 healthy athletic participants from 6 to 29 years, the 3D-MOT exercise has recently demonstrated shared predictive validity with other cognitive tests used for concussion assessment such as the SCAT3 and the King-Devick test.^28^ In addition, a preliminary study in a paediatric population with and without mTBI suggested that 3D-MOT could be beneficial for stimulating recovery and informing return to activity decisions.^29^ Given its relevance to perceptual-cognitive abilities in sport, its predictive power for performance in socially relevant tasks and the recent evidence for concussion assessment, 3D-MOT was tested for the evaluation and management of sport concussion in professional athletes in the context of this study. The main objective of this study was to introduce the 3D-MOT technique for concussion evaluation and return to play in professional athletes.

## Methods

### Participants

Fifty-nine professional athletes (24.78±5.15 years, table 1) from national French leagues (Ligue Nationale de Rugby, Ligue de Football Professionnel, Ligue Nationale de Handball, Fédération Française de Judo) participated in the study. Participants were referred by the sport’s club physician following what was suggested to be a concussion on the playing field (eg, HIA 1 protocol). The concussion was confirmed by the neurologist during the first evaluation within 48 hours following the injury (table 2).

**Table 1:** Characteristics of the professional athlete participants

Athletes	Number	Percentage
Gender		
Male	48	81
Female	11	19
Sports		
Rugby	50	85
*Forwards*	*31*	*62*
*Backs*	*19*	*38*
Judo	6	10
Soccer (Football Association)	1	2
Football	1	2
Handball	1	2
Athletic level		
International	39	66
National	20	34
History of injury		
Previous concussion	44	75
Mean number ±SEM	2.75±0.33 concussions	
Max/min	12/1 concussions	
No previous concussion	15	25
Neurologist evaluation		
Mean delay 48 hours—return to play	20.61±2.47 days	
Max/min delay	84/2 days	
Mean delay before return to play	26.08±2.78 days	

**Table 2:** History of the injury

mTBI athletes	Number	Percentage
Cause of injury		
Hits	54	92
Falls	5	8
Occurence of injury		
Game	46	78
Training	13	22
Admission Cantu grade		
Severe (3)	46	78
Moderate (2)	9	15
Mild (1)	2	3
Unknown	2	3
Admission AAN grade		
3	14	24
2	43	73
1	2	3
LOC		
Yes	16	27
No	43	73
Antero-amnesia		
No	32	54
≤1 min	8	14
≥1 min	19	32
Retro-amnesia		
No	48	81
≤1 min	5	8
≥1 min	6	10
Initial symptoms		
Headache	47	80
Fatigue	40	68
Pressure in head	31	53
Sonophobia/photophobia	30	51
Neck pain	30	51
Difficulty concentrating/remembering	29	49
Balance problems	24	41
Drowsiness	23	39
Nausea—vomitting	22	37
Trouble falling asleep	21	36
Visual problems	19	32
Duration of symptoms		
Mean duration ±SEM	9.88±1.56 days	
Max/min duration	*56 days/30* min	

AAN, American Academy of Neurology; LOC, Loss of consciousness; mTBI, mild traumatic brain injury.

### Evaluation tests

#### Three-dimensional multiple-object tracking

**Figure 1:**
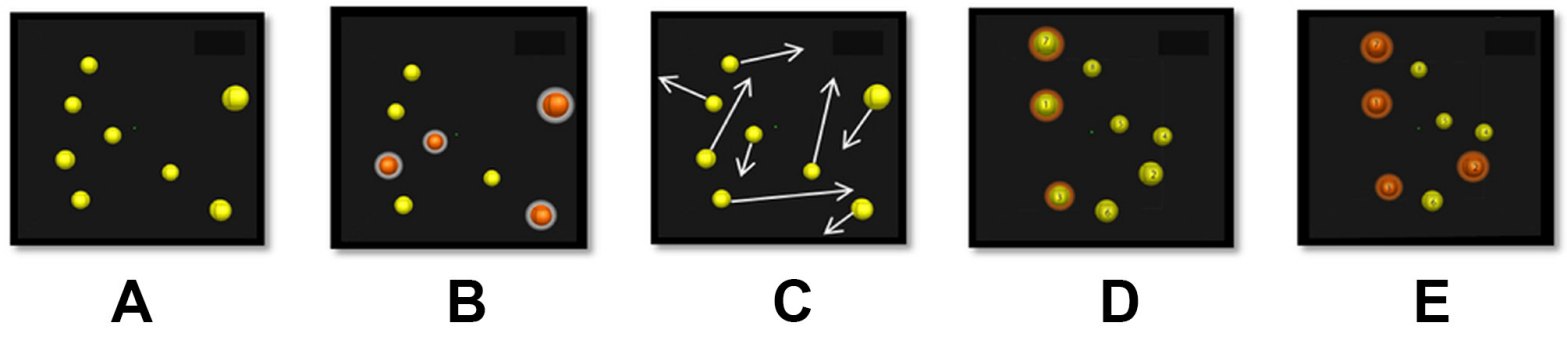
NeuroTracker ‘CORE’ mode: (A) presentation, (B) target identification, (C) displacement, (D) user response and (E) feedback.

The commercial version of 3D-MOT called NeuroTracker (CogniSens Inc.) was used to assess the perceptual-cognitive state of the participants. The ‘CORE’ mode of the NeuroTracker was displayed on a 65’’ 3D-TV (figure 1). The exercise required participants to track four targets among eight spheres projected within a virtual cube space for 8 s (one trial), controlling for a visual angle of 45°. A detailed description of the methodology can be found in previous studies.^21 30^ A typical session, based on a staircase procedure, required approximately 6 min which consisted of a total of 20 trials. Three consecutive 3D-MOT sessions were completed during each evaluation for a total of 20 min.

#### Standardized Assessment of Concussion

The SAC (SCAT 3) was used to assess athletes’ multiple mental components such as orientation, immediate memory, concentration and delayed memory.^31^ The SAC test contains a series of questions which take approximately 5 min to administer and is scored out of 30 points (total score). A standard neurological screening is also included to assess LOC, retrograde and post-traumatic amnesia, deficits in strength, sensation and coordination that may result from a concussion. Three equivalent alternate forms of the test were used at baseline, 48 hours and RTP to minimise practice effects from additional administration.

#### Modified Balance Error Scoring System

The M-BESS is a modified version of the BESS, a standard balance assessment to evaluate static balance.^15 32–34^ It consists of three stances: double-leg stance (feet together), single-leg stance (standing on the non-dominant leg) and a tandem stance (non-dominant foot behind the dominant foot in a heel-to-toe fashion). The number of errors in deviations from the proper stance (eg, moving hands off of iliac crests, opening eyes, a step/stumble or fall, abduction or flexion of the hip beyond 30°, lifting forefoot or heel off testing surface) were substracted from a score of 10 for each stance. Only one error was counted when multiple errors occurred at the same time. The maximum total score for each testing condition was 30 (total score). The test was conducted on a firm surface.

### Procedure

Previous French rugby consensus statement on concussion in sport established that a consultation with a neurologist could help return to sports under optimal conditions to prevent recurrent concussions.^35 36^ Moreover, evidence has shown that consultation within 4 days following the injury could help diminish the duration of post-concussion syndrome due to a more thorough follow-up and support of the concussed athlete.^35–37^ In this regard, athletes who potentially sustained a concussion were sent to an independent neurologist and underwent the standard procedure developed in collaboration with the medical staff of the sport clubs that participated in the study (table 3). Two main evaluations were conducted by the same neurologist, one within 48 hours following the injury (48 hours) and the second when deciding the RTP. Note that 32 professional rugby players from the present sample were also tested on SAC and M-BESS tests in pre-season by the neurologist, and therefore, a baseline score had been established.

**Table 3:** Study protocol for concussion management adapted from ref.^36^

Steps	Concussion management protocol
1	Immediate removal from the field and total rest for the patient with concussion
2	CT scan or brain magnetic resonance imaging (optional)
3	First consultation with the neurologist within 48 hours following the injury including
3a	Confirmation of the diagnosis
3b	Cantu classification for concussion severity
3c	symptoms’ assessment, performance on SCAT and NeuroTracker evaluation
4	Return to exercising in stages once post-concussive symptoms at rest have disappeared:
4a	Walking or biking
4b	Individual running
4c	Training without contact
5	Second consultation with the neurologist in the absence of recurrent symptoms during exercising
5a	Confirmation of the recovery based on cognitive evaluation (SCAT and NeuroTracker)
5b	Clear for return to play or follow-up visit

SCAT, Sport Concussion Assessment Tool.

### Analysis

Statistical analysis was performed on the main three dependant variables: 3D-MOT speed threshold, SAC and M-BESS scores. The normality of distribution in each case was controlled using asymmetry, skewness and Shapiro-Wilk tests. In case of normal distribution, analysis of variance (ANOVA) was used including pairwise comparisons using Bonferroni corrections. Post-hoc comparisons were performed using Student’s t tests. Levene and Mauchly tests were also performed to ensure that the homogeneity of variance and the sphericity, respectively, were not violated (p>0.05). As SAC and M-BESS scores resulted in being not normally distributed, non-parametric tests were employed to analyse these variables. Eta-squared (η^2^) or Cohen’s d values were reported to provide information about the magnitude of the effect. Statistical analyses were performed using IBM SPSS statistics V.23.

#### Three-dimensional multiple-object tracking

A repeated-measures ANOVA was employed to compare 3D-MOT *Sessions* (1, 2, 3 and 4, 5, 6) and 3D-MOT *Evaluations* (48 hours, RTP). An additional analysis was processed including a sample of healthy professional athletes (HP) considered as normative data.^22^ A repeated-measure ANOVA was conducted with the within-subject factors 3D-MOT *Session* (1, 2, 3 and 4, 5, 6), 3D-MOT *Evaluations* (48 hours, RTP) and the between-subject factor *Groups* (mTBI, HP). Moreover, logarithmic regression functions were fitted on speed thresholds of evaluations at 48 hours (sessions 1, 2 and 3) and RTP (sessions 4, 5 and 6) for each group. A repeated-measure ANOVA was performed with the within-subject factor *Evaluations* (48 hours, RTP) and the between-subject factor *Groups* (mTBI, HP) to compare learning rates on speed thresholds.

#### Standardized Assessment of Concussion and Modified Balance Error Scoring System

First, test scores were analysed using Wilcoxon tests with the repeated factor *Evaluations* (48 hours, RTP). Second, another analysis was performed on a smaller sample of concussed players (n=32) because pre-season baseline measures were available. Friedman tests were employed on SAC and M-BESS scores with the repeated factor *Evaluations* (baseline, 48 hours, RTP). Post-hoc comparisons were processed using Wilcoxon tests.

#### Correlations

Spearman rank correlation coefficient was used to investigate the possible associations between NeuroTracker, SAC and M-BESS scores as well as the number of total symptoms, symptoms duration, delay before returning to play or Cantu grade.

## Results

### Three-dimensional multiple-object tracking

**Figure 2:**
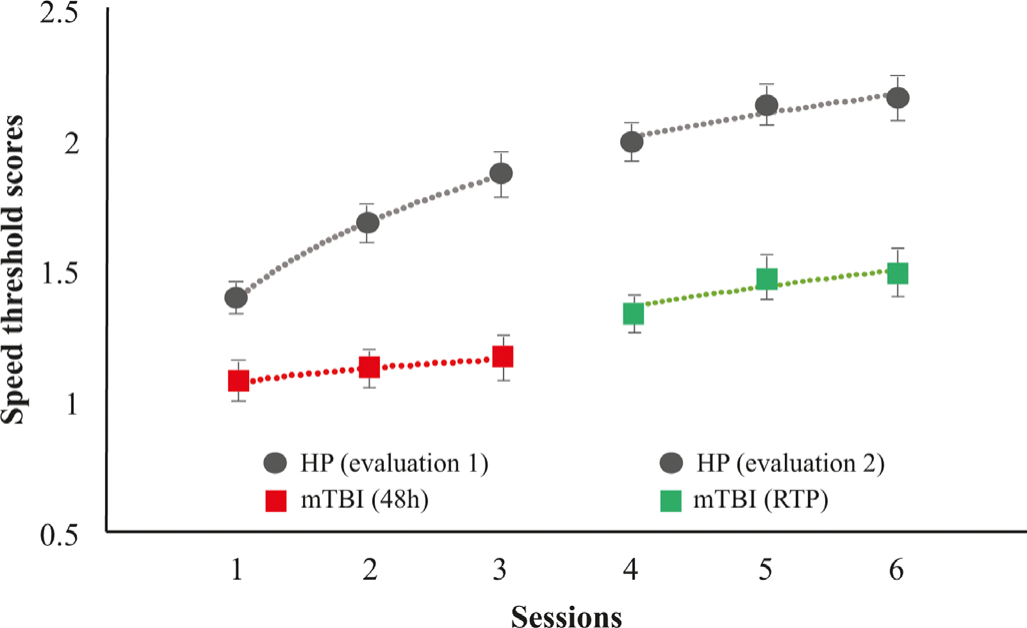
Comparison of the three-dimensional multiple-objecttracking scores between concussed and healthy athletes throughout a first evaluation (48 hours post injury) and a second evaluation (RTP). HP, healthy professional athletes; mTBI, mild traumatic brain injury; RTP, returnto play.

The analysis demonstrated a significant main effect of *Evaluations* (F[1,58]=86.948, p<0.001, η^2^=0.600) with a moderate to strong effect size (d=0.772) indicating a better performance on 3D-MOT at RTP compared with 48 hours post injury (figure 2). There was also a significant main effect of 3D-MOT *Sessions* (F[2,116]=10.738, p<0.001, η^2^=0.156). The interaction between *Evaluations* and 3D-MOT *Sessions* was non-significant (F[2,116]=0.233, p=0.792, η^2^=0.004), revealing that the improvement across 3D-MOT sessions was not significantly different at RTP and 48 hours. The comparison between mTBI and healthy professional athletes resulted in a strong significant difference between *Groups* (F[1,117]=61.350, p<0.001, η^2^=0.344) corroborating the impact of the injury on 3D-MOT performance. Moreover, the significant interaction between *Evaluations* × *Sessions* × *Groups* (F[2,234]=4.053, p=0.019, η^2^=0.033) confirmed that concussed athletes had lower performance in processing and learning the 3D-MOT task compared with healthy professionals who improved better and faster on the exercise between evaluations at 48 hours and RTP. Learning rates analysis demonstrated a significant interaction between *Evaluations* × *Groups* (F[2,117]=6.717, p=0.011, η^2^=0.054) revealing slower learning rates on 3D-MOT in concussed compared with healthy athletes. Additional analysis demonstrated a significant difference in slopes between mTBI and healthy professionals at 48 hours (t[117]=3.867, p<0.001) but not at RTP (t[117]=0.350, p=0.727). This stems from the fact that the learning function within the 48 hours of concussion is severely affected compared with healthy pros (figure 2).

### Standardized Assessment of Concussion and Modified Balance Error Scoring System

**Figure 3:**
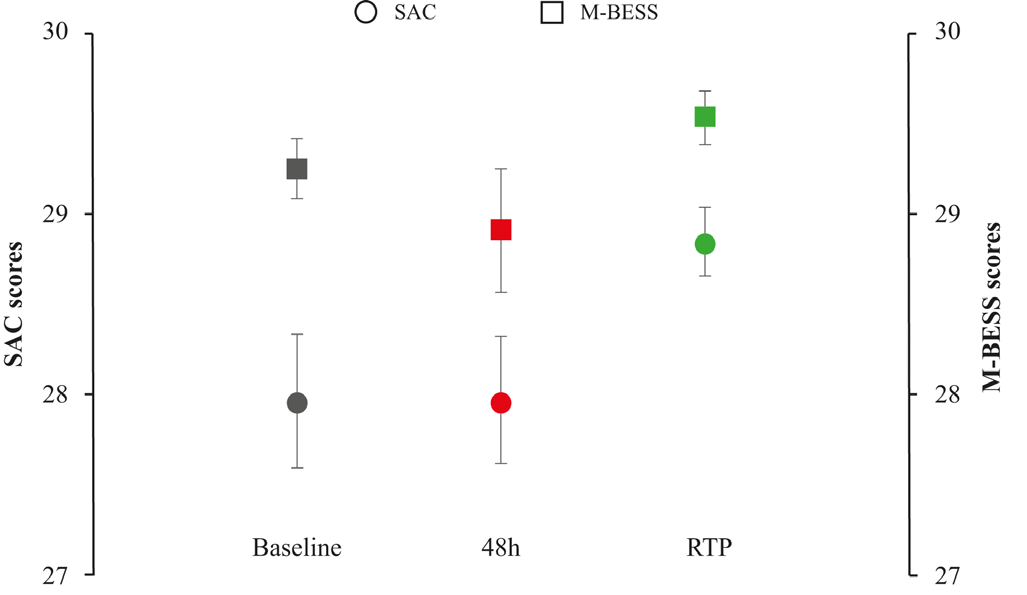
Standardized Assessment of Concussion (SAC) and Modified Balance Error Scoring System (M-BESS) scores across evaluations before (baseline), 48 hours and at return to play (RTP) time following concussion.

First results demonstrated a significant difference in performance score with moderate effect size between evaluations at 48 hours and RTP in SAC (Z=−3.982, p<0.001, d=−0.635) as well as M-BESS (Z=−3.433, p=0.001, d=−0.516) tests (figure 3). However, another repeated-measure analysis within the same group of athletes and including baseline evaluation only showed a tendency towards significance in SAC scores between evaluations at baseline, 48 hours and RTP (χ^2^[2]=5.766, p=0.056). Post-hoc comparisons demonstrated no significant difference in SAC scores between evaluations at baseline and 48 hours (Z=−0.87, p=0.931, d<−0.1) which cannot indicate the presence of cognitive problems caused by the concussion. Significant differences with moderate effect size were seen between evaluations at 48 hours and RTP (Z=−2.489, p=0.013, d=0.550) as well as evaluations at baseline and RTP (Z=−2.249, p=0.025, d=0.536). The analysis on M-BESS scores demonstrated no significant difference between evaluations at baseline, 48 hours and RTP (χ^2^[2]=2.103, p=0.349).

### Correlations

First, there were weak but significant positive correlations between 3D-MOT and SAC scores (r_s_=0.282, p=0.031), between 3D-MOT and M-BESS scores (r_s_=0.368, p=0.004) as well as between SAC and M-BESS scores (r_s_=0.301, p=0.020) at 48 hours which indicated that these three measures of evaluation shared some predictive validity related to concussion (table 4). Importantly however, only 3D-MOT scores at 48 hours correlated negatively with the number of total symptoms (r_s_=−0.301, p=0.020). Although the correlation was weak, it suggests an association between lower performance and higher number of symptoms and vice versa. On the other hand, there were no significant correlations between symptoms and SAC or M-BESS scores at 48 hours (p<0.05). Not surprisingly, the total number of symptoms correlated positively and moderately with symptom duration (r_s_=0.684, p<0.001), delay before returning to play (r_s_=0.542, p<0.001) and Cantu grade (r_s_=0.364, p=0.005).

**Table 4:** Spearman rho correlations

	MOT3D- 48 hours	MOT3D -RTP	SAC48 hours	SACRTP	M-BESS48 hours	M-BESSRTP	TS	SD	RTPdelay	Cantu grade	History of mTBI
3D-MOT 48 hours	1										
3D-MOT RTP	0.770***	1									
SAC 48 hours	0.282*	0.187	1								
SAC RTP	0.18	0.227	0.343**	1							
M-BESS 48 hours	0.368**	0.174	0.301*	0.106	1						
M-BESS RTP	0.184	0.146	0.121	-0.049	0.506***	1					
TS	–0.301*	–0.159	–0.16	–0.071	–0.139	0.109	1				
SD	–0.089	–0.029	–0.056	–0.13	–0.17	0.01	0.684***	1			
RTP delay	–0.191	–0.148	–0.1	–0.201	–0.097	0.013	0.542***	0.591***	1		
Cantu grade	–0.062	–0.061	0.011	–0.024	0.101	0.033	0.364**	0.522***	0.472***	1	
History of mTBI	–0.036	0.036	0.271*	0.108	0.288*	0.228	0.11	0.134	0.199	0.21	1

*p<0.05, **p<0.01, ***p<0.001.

D-MOT, three-dimensional multiple-objecttracking; M-BESS, Modified Balance Error Scoring System; mTBI, mild traumatic brain injury; RTP, return to play; SAC, Standardized Assessment of Concussion; SD, symptoms duration; TS, total symptoms.

## Discussion

This study assessed the usefulness of a perceptual-cognitive 3D-MOT exercise for the evaluation and RTP guidance of sport-related concussion. The 3D-MOT was found to be a relevant perceptual marker to help monitor sport-related concussion. Importantly, 3D-MOT performance was correlated with the total number of symptoms, SAC and M-BESS scores at 48 hours. However, results on SAC and M-BESS tests demonstrated less relevance for monitoring concussion and correlation to symptoms in the present study using professional athletes.

### Perceptual-cognitive impairments following sport-related concussion

The main finding revealed the negative impact of mTBI on both processing and learning the perceptual-cognitive 3D-MOT task at 48 hours compared with RTP and compared with normative scores in healthy professionals. The results were congruent with preliminary works in a general population (n=485) where three sessions of 3D-MOT were shown to be sensitive to mTBI status compared with healthy individuals or individuals with a history of mTBI.^38^ The 3D-MOT performance was significantly enhanced at RTP compared with 48 hours post -injury which supports the usefulness of this tool for RTP guidance.

Importantly, learning rates were strongly affected at 48 hours post injury which is again consistent with recent findings in a paediatric mTBI population where processing and learning were disrupted during the first sessions of exposure to the task compared with healthy children.^29^ The present result obtained in professional athletes is of particular interest knowing that this population is characteristic of better processing and learning on such a task.^22^ It emphasises the fact that athletes’ perceptual-cognitive capacity is very limited during the early days following the injury and reinforces the fact that one should be cautious when quickly returning to activities involving dynamic visual processing following mTBI. This result is also consistent with previous evidence showing cognitive problems following concussion.^39^

Another important finding in this study revealed that 3D-MOT performance was significantly correlated to the number of total symptoms at 48 hours. Although the correlation was weak, it suggests that speed thresholds scores were higher when the symptoms were lower and vice versa. Other correlational analysis demonstrated a link between 3D-MOT, SAC and M-BESS at 48 hours following the injury. These results support previous evidence showing predictive validity of 3D-MOT with other concussion assessment cognitive tests such as the SCAT3 and King-Devick test.^28^

### SAC and M-BESS tests are weak predictors of the RTP

The results of the present study revealed weak usefulness of the SAC and BESS tests in the RTP guidance when reviewed comparatively with the baseline scores. In fact, it was difficult to determine if the difference observed between scores at 48 hours and at RTP was due to a sensitivity to the injury or simply a test–retest effect.

This is not surprising considering that previous studies have emphasised the limitations of the M-BESS for balance deficits assessment including insufficient repeatability, poor reliability, fatigue effects, influences from musculoskeletal injuries and learning effects.^13 14^ Reliability of the test can range from poor to good and has been shown to be sensitive to concussion assessment only within the first three days post injury.^15 40^ Importantly, poor inter-rater reliability due to the subjective nature of the scoring system and important practice effects have also been pointed out.^15^ Recently, the BESS test has been identified with a high rate of false positives (62.5%) when baseline scores were compared with scores obtained at a later date.^41^ This former study also determined a high rate of false positives with the SAC test (27.1%).

Similarly, the usefulness of the SAC test to evaluate cognition and memory^42^ while monitoring concussion in professional athletes was weak considering the present results. Previously, the literature has shown that although it has good sensitivity and specificity,^31 43^ scores consistently returning to baseline after 48 hours or after three trials demonstrates a practice effect that may result in misinterpretation and poor management of the rehabilitation following concussion.^16 44^ Another study reported small to moderate effect sizes for the SAC and BESS at 24 hours following the injury that became non-significant at day 8 (SAC) and day 15 (BESS) post concussion.^17^ This former study also suggested that concussion symptoms were the most sensitive component of the SCAT3 compared with the cognitive (SAC) and motor (BESS) subsets. In the present study, the SAC and M-BESS tests were not correlated with the number of total symptoms. The results confirmed that SAC and M-BESS are not helpful in monitoring concussion and suggests that reliable tools other than SAC and M-BESS are still needed to help the management of sport-related concussion.

### Perceptual-cognitive assessment in sport-related concussion

The present study emphasised the fact that concussion assessment tools should consider some critical components requested to process dynamical environment, such as perceptual-cognitive screening. Perceptual-cognitive skills are required while processing visual dynamic scenes (eg, walking into a crowd, avoiding collision, or anticipating and decision-making in sport). In this regard, the 3D-MOT technique relies on an attentional task requiring the tracking of multiple moving targets (ie, MOT) with increasing speed which addresses the dynamic components of the living environment. Two strategies are typically employed by the subject to process the MOT task. The first is a ‘centre-looking’ strategy that consists of grouping the targets into a single object while looking closer to the centre of the object formed by the targets.^45^ The other is a ‘target-looking’ strategy where the subject would saccade from target to target. Other evidence has shown that participants often engaged in both strategies by switching their gaze from the centre to the targets and so on.^46^ Visual search strategies involved during MOT could be closely linked to those applied by sport experts during the process of extracting visual information from their action-rich environment. Recently, more evidence has correlated mTBI with impaired attentional tracking.^47 48^ In addition, the virtual testing environment involves stereoscopic vision which is critical when interacting in the real world.^49^ The technique also integrates a large visual field of view which is similar to most sporting scenarios.^50^ In the future, it will be essential to compare the 3D-MOT task to other perceptual-cognitive or computerised neurocognitive tests (eg, King-Devick test) to measure the efficacy of such techniques in monitoring concussion.

### Limitations

Despite the relevance of the perceptual-cognitive 3D-MOT technique in monitoring sport-related concussion, the study presents some limitations. For instance, there were no 3D-MOT baseline tests performed on the present cohort of athletes. Although recent evidence tends to show that there is no clear advantage of using a baseline-referenced approach over a norm-referenced approach.^51^ Moreover, this should have a limited impact on the results since the sample of healthy professionals closely matched the injured professionals in terms of sport, league-level (eg, Ligue Nationale de Rugby), age and experimental design. Another drawback of the present methodology is that the 3D-MOT assessment took about 20 min to complete (which included three consecutive sessions for learning assessment purposes). In the future, the predictive value of a shortened 3D-MOT assessment (eg, one session) should be evaluated and compared with other perceptual-cognitive tests. However, this modification to the procedure would not include an assessment of learning rates which has clearly shown to be an advantage over other techniques in the present and previous concussion studies.^29^

## Conclusion

For the first time, this study presents the unique role of a perceptual-cognitive 3D-MOT exercise to monitor sport-related concussion. This technique seems to possess some of the requirements needed to appropriately respond to everyday world and sport specific demands. Future studies should compare its efficacy to other perceptual-cognitive techniques, evaluate if reducing 3D-MOT assessment time can preserve its potential towards monitoring concussion and should also test its training value for post-concussion rehabilitation.
